# Fungal zinc metabolism and its connections to virulence

**DOI:** 10.3389/fcimb.2013.00065

**Published:** 2013-10-14

**Authors:** Charley C. Staats, Lívia Kmetzsch, Augusto Schrank, Marilene H. Vainstein

**Affiliations:** ^1^Centro de Biotecnologia, Universidade Federal do Rio Grande do SulPorto Alegre, Brazil; ^2^Departamento de Biologia Molecular e Biotecnologia, Universidade Federal do Rio Grande do SulPorto Alegre, Brazil

**Keywords:** zinc ZIP transporters, zinc metabolism, zinc deprivation, ZAP transcription factor, fungal virulence

## Abstract

Zinc is a ubiquitous metal in all life forms, as it is a structural component of the almost 10% of eukaryotic proteins, which are called zinc-binding proteins. In zinc-limiting conditions such as those found during infection, pathogenic fungi activate the expression of several systems to enhance the uptake of zinc. These systems include ZIP transporters (solute carrier 39 family) and secreted zincophores, which are proteins that are able to chelate zinc. The expression of some fungal zinc uptake systems are regulated by a master regulator (Zap1), first characterized in the yeast *Saccharomyces cerevisiae*. In this review, we highlight features of zinc uptake and metabolism in human fungal pathogens and aspects of the relationship between proper zinc metabolism and the expression of virulence factors and adaptation to the host habitat.

## Introduction

Zinc is fundamental for all domains of life, as it composes the catalytic and structural center of a large array of proteins. Thus, the “zinc quota,” defined as the cellular zinc content required for optimal growth (Outten and O'halloran, 2001), must be finely tuned. There is a huge variation in the number of zinc atoms per cell in different organisms (10^5^ for *Escherichia coli*, 10^7^ for yeast, and 10^8^ for mammalian cells). However, given cell size variation, zinc concentration is kept in close limits (0.1–0.5 mM) (Eide, 2006). The zinc quota is maintained by the activity of specific membrane transporters or by zinc-binding proteins that mediate zinc uptake or storage. Zinc-depleting conditions are known to reduce fungal growth (Lulloff et al., 2004) and evidence suggests that host cells employ sequestration of zinc to hamper fungal development. This is exemplified by the reduced zinc levels in macrophages infected with *Histoplasma capsulatum* (Winters et al., 2010), and neutrophils infected with *Cryptococcus neoformans* enhance the production of calprotectin, a zinc-binding protein (Mambula et al., 2000). However, excess cellular zinc can generate an imbalance in oxidative metabolism (Pagani et al., 2007). Indeed, macrophages have developed a strategy to kill phagocytosed bacterial cells by zinc overload in the phagosomal environment and the consequent generation of high levels of reactive oxygen species (ROS) in the invading microorganisms (Botella et al., 2011). Here, we focused on the proteins involved in zinc metabolism in the fungal pathogens *Aspergillus fumigatus*, *Candida albicans, C. neoformans*, and *C. gattii* as compared to the well-characterized zinc metabolism-associated proteins from *S. cerevisiae*. Moreover, a critical appraisal on the participation of zinc metabolism and associated proteins in the establishment of fungal infections is presented.

## Zinc-associated biological processes in fungi

**Figure 1:**
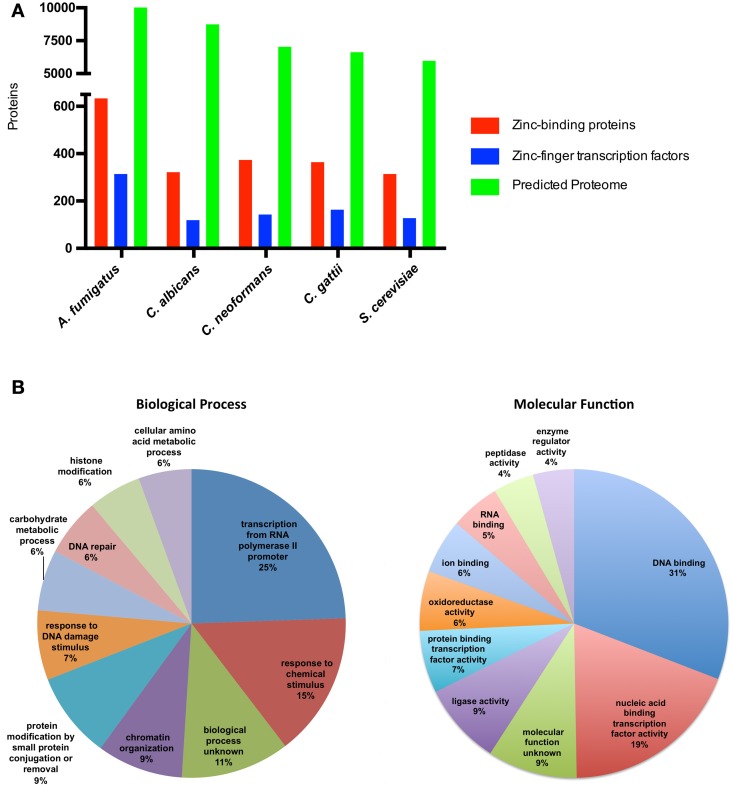
**Zinc-binding proteins associated biological processes in fungi. (A)** The number of zinc finger transcription factors and zinc-binding proteins compared to the total proteome from fungi. The predicted proteomes of *A. fumigatus, C. albicans, C. neoformans, C. gattii*, and *S. cerevisiae* were retrieved from their respective genome assemblies in NCBI (Accession numbers GCA_000002655.1, GCA_000182965.2, GCA_000149245.2, GCA_000185945.1, GCA_000146045.2, respectively). The protein sequences were submitted to the UFO webserver (Meinicke, 2009) for functional profiling. All proteins with the Gene Ontology annotation “zinc ion binding” (GO:0008270) were considered to be zinc-binding proteins. **(B)** The 311 yeast proteins annotated as zinc-binding proteins in the Yeast Genome Database were submitted to GO-slim analysis (Cherry et al., 2012). The 10 most abundant biological process (left graph) and molecular function (right graph) associations were considered, and the fraction of proteins in each class is depicted.

There is little labile zinc inside cells; the estimated concentration of zinc in cells is in the picomolar to the nanomolar range (Eide, 2006), and it is assumed that virtually all cellular zinc is associated with zinc-binding proteins. Bioinformatic analyzes to evaluate the presence of canonical sequences related to zinc binding have revealed that 5–6% of the predicted proteomes of prokaryotes consist of zinc-binding proteins, while this proportion reached 9% in eukaryotic proteomes (Andreini et al., 2009). Using the fungal model *S. cerevisiae*, with a manually curated annotation of the genome and considerable biochemical information (Cherry et al., 2012), it is possible to infer the fraction of the proteome that can bind zinc. In fact, analyzes of predicted gene products from different organisms using the term “zinc ion binding” as a query of Gene Ontology databases revealed that some fungal species have a proportion of zinc-binding proteins that corresponds to ~5% of the proteome (Figure 1). Considering only the zinc-binding proteins from *S. cerevisiae*, a large proportion of these proteins (25%) are associated with biological processes related to transcriptional regulation. An even higher proportion of these proteins have the ability to bind DNA as accessed by Gene Ontology analysis (Figure 1). These numbers and the accumulation of a significant number of experimental reports point to the central role of zinc in gene expression regulation.

Consistent with the plethora of zinc-binding proteins associated with the regulation of gene expression in *S. cerevisiae*, a large number of these zinc-binding proteins consist of zinc finger transcription factors, the largest family of transcriptional regulators. Analysis of the Fungal Transcription Factor Database (Park et al., 2008) revealed that fungal species are characterized by a high diversity of zinc finger transcription factors, ranging from 116 in *C. albicans* to 311 in *A. fumigatus* (Figure 1). The vast majority of these transcription factors belong to the widely phylogenetically distributed Zn2Cys6. In *S. cerevisiae*, members of this family are involved in the regulation of several biological processes, including sugar and amino acid metabolism, nitrogen utilization, mitosis, and meiosis (Macpherson et al., 2006). However, other biological processes are associated with zinc-binding proteins. This is consistent with the enzymatic activity present in some zinc-binding proteins, including ligase, peptidase, and oxidoreductase activities (Figure 1). Yet in *S. cerevisiae*, the Cu/Zn superoxide dismutase and the alcohol dehydrogenase (Adh1) proteins are the most abundant zinc-binding proteins (Eide, 2006).

Some zinc-binding proteins are also involved in fungal virulence. Superoxide dismutases (Sods) are the central enzymes in fungi associated with the detoxification of ROS generated by host cells during host-pathogen interactions (Huang et al., 2009). In this view, specific Sods from pathogenic fungi are assumed to be virulence determinants. *C. albicans* expresses six superoxide dismutase isoforms (Sod1—Sod6), four of which are annotated as copper/zinc-dependent enzymes (Sod1, Sod4, Sod5, and Sod6) (Frohner et al., 2009). Functional analysis has revealed that Sod1, Sod4, and Sod5 are necessary for the proper detoxification of ROS by *C. albicans*, as SOD-null mutants displayed growth defects in the presence of ROS-generating compounds. In addition, these mutants displayed increased susceptibility to macrophage killing and reduced virulence (Hwang et al., 2002; Martchenko et al., 2004; Frohner et al., 2009). The *A. fumigatus* genome contains four genes encoding Sods, two of which are annotated as copper/zinc-dependent (Sod1 and Sod4). *A. fumigatus* cells lacking the *SOD1* gene are hypersensitive to menadione, a ROS generating agent, but the virulence of cells lacking this gene is not affected (Lambou et al., 2010). In *C. neoformans*, two Sod-encoding genes have been described. The *SOD1* gene encodes a copper/zinc-dependent Sod required for full virulence in animal models of cryptococcosis and for survival inside macrophages (Cox et al., 2003).

Zinc-binding metalloproteases have also shown to be involved in virulence. Distinct species of pathogenic fungi secrete proteases during the infection. These proteases are classified into aspartic proteases, serine proteases, and metalloproteases (Yike, 2011). The deuterolysin (M35) family of metalloproteases is characterized by the presence of two zinc-binding histidines and a catalytic glutamate in their catalytic centers (Markaryan et al., 1994). The roles of metalloproteases secreted by pathogenic fungi are largely associated with tissue degradation. This is evident for the Mep3 metalloprotease from *Microsporum canis* (Brouta et al., 2001). The ADAM proteases (from A Disintegrin And Metalloproteinase) belongs to the M12 family of metalloproteases according to the MEROPS database (Rawlings et al., 2012). These proteins are produced as pro-enzymes that must be secreted and activated prior to performing their biological functions. ADAM proteases have been implicated in several aspects of cell biology including adhesion, migration, proteolysis, and signaling (Edwards et al., 2008). The presence of two copies of putative ADAM coding sequences in the genome of *A. fumigatus* indicates a possible contribution for this family in virulence in this fungus. However, no functional characterization was performed yet to evaluate whether ADAM proteases can be associated to virulence in *A. fumigatus.*

## Fungal zinc uptake

Fungal cells must acquire zinc for proper development during their life cycle, even when they are saprophytes or during the infection process. To hamper pathogen growth, mammalian hosts typically reduce the levels of free zinc and other metals (Kehl-Fie and Skaar, 2010). The concentration of zinc in human tissues varies dramatically, ranging from 10 μ g/g (lungs) to 83.2 μ g/g (liver). In body fluids, the zinc concentration ranges from 0.2 to 8.7 μ g/mL (Lech and Sadlik, 2011). Thus, pathogenic fungi have developed efficient strategies to uptake zinc to overcome the limits imposed by host.

The initial characterization of zinc transport mechanisms in fungi was done in *S. cerevisiae* and revealed the central role of the ZIP (Zrt-, Irt-like protein) family of zinc transporters (Eide, 2006). The name of this family, also known as SLC39 (solute carrier 39), refers to the first members to be functionally characterized, the *S. cerevisiae* zinc transporters Zrt1 and Zrt2 and the *Arabidopsis thaliana* iron transporter Irt1 (Eide, 2004). ZIP family transporters are associated with zinc transport into the cytoplasm across cellular membranes, either from the extracellular space or from within organelles. ZIP transporters are characterized by eight putative transmembrane regions, and the amino- and carboxyl-termini are often located on the extracellular or luminal side of membranes (Eide, 2004). A histidine-rich region present between transmembrane regions three and four is necessary for zinc selectivity, as demonstrated for the TjZNT1 ZIP transporter from the nickel hyperaccumulator plant *Thlaspi japonicum* (Nishida et al., 2008).

The *S. cerevisiae* Zrt1 is a high-affinity zinc transporter that is expressed when cells are cultivated in low-zinc media (Zhao and Eide, 1996a), while the low-affinity transporter Zrt2 mediates the uptake of zinc, cooper, and iron (Zhao and Eide, 1996b). Additional non-specific zinc transporters are also associated with zinc uptake, as *zrt1/zrt2* double-mutants are capable of growing in low-zinc conditions (Zhao and Eide, 1996b). These transporters include the low-affinity iron transporter Fet4 and the phosphate transporter Pho84, which mediate the uptake of zinc by phosphate chelation of this metal (Waters and Eide, 2002; Jensen et al., 2003). Inside cells, zinc is shuttled to different compartments, including the nucleus, endoplasmic reticulum and vacuole, by the activity of specific transporters not related to the ZIP family (Eide, 2009). Zrt3 is a ZIP transporter found in the membranes of vacuoles that accumulate zinc, the so-called “zincosomes.” The function of Zrt3 is associated supplying zinc to the cytoplasm from zincosomes (Simm et al., 2007).

The number of ZIP genes in the genomes of *A. fumigatus*, *C. albicans*, *C. neoformans*, and *C. gattii* ranges from four to nine. Genome annotation based evidences suggest that many of these transporters show high similarity to *S. cerevisiae* Zrt1 or Zrt2 proteins, suggesting that both low- and high-affinity zinc uptake systems exist in these pathogenic fungi. In *A. fumigatus*, three ZIP zinc transporters have been characterized. The expression of *zrfA* and *zrfB* genes is activated by low levels of zinc or iron. In addition, the expression of these transporters in response to zinc deprivation occurs mainly in an acidic environment. Null mutants for these genes, as well as double mutants lacking both genes, showed a reduced ability to grow under zinc deprivation (Vicentefranqueira et al., 2005). Further analysis showed that the expression of these genes is under control of the pH homeostasis regulator PacC (Amich et al., 2009). A third zinc transporter from *A. fumigatus*, which is encoded by the gene *zrfC*, is expressed during zinc-deprivation conditions when the fungus is grown in alkaline pH conditions. Null mutants of this gene are severely reduced in their ability to grow during zinc deprivation (Amich et al., 2010). In *C. gattii*, zinc deprivation by the chelator N,N,N,N-tetrakis(2-pyridylmethyl)ethylenediamine (TPEN) induced the expression of the putative zinc transporters encoded by genes *ZIP1*, *ZIP2*, and *ZIP3* (Schneider et al., 2012).

Some fungal species possess additional mechanisms to sequester zinc from host cells and tissues in a process analogous to iron chelation by secreted siderophores. *C. albicans* secretes the antigenic protein Pra1, a zinc-binding protein that is able to scavenge zinc from tissues invaded by the fungus. Moreover, molecular docking experiments revealed that Pra1 could interact with the zinc transporter Zrt1. Pra1 participates in proper endothelial colonization by *C. albicans* (Citiulo et al., 2012) and is associated with evasion of immune cells (Luo et al., 2011; Soloviev et al., 2011). In addition, orthologs of the gene encoding Pra1 are found in diverse fungal pathogens (Citiulo et al., 2012) including *A. fumigatus* (the zinc-regulated *aspf2* gene (Amich et al., 2010). However, no orthologs could be found in *C. neoformans* or *C. gattii.* These zinc-sequestering genes are generally clustered with Zrt1 orthologs in these fungi in a highly syntenic fashion (Citiulo et al., 2012), representing a conserved mechanism for zinc acquisition during host-fungal interactions.

## Effects of zinc deprivation on fungal cells

Zinc chelation is able to reduce fungal growth in both rich and defined media (Lulloff et al., 2004). In fact, zinc chelation is assumed to occur during infection and is an important strategy developed by immune cells to hamper pathogen growth (Corbin et al., 2008). Zinc restriction by host cells is achieved by lowering metal availability via the activity of the host zinc transporters or the expression of zinc-binding proteins. An example of a zinc-binding protein that is expressed to reduce the bioavailability of zinc is calprotectin, a member of the S100 family of metal-binding proteins (Goyette and Geczy, 2011). Calprotectin was found to reduce the growth of diverse fungal species *in vitro* (Lulloff et al., 2004). Moreover, this protein is produced by neutrophils in order to reduce the development of *A. fumigatus* (McCormick et al., 2010), *C. albicans* (Urban et al., 2009), and *C. neoformans* (Mambula et al., 2000). Neutrophils are able to kill invading pathogens by phagocytosis, secreting anti-microbial molecules and forming neutrophil extracellular traps (NETs). NET formation is derived from a distinct mechanism of cell death that is characterized by the loss of intracellular membranes and further structural derange of the plasma membrane. As a result of this loss of membrane functionality, NETs are composed of nucleosomes and a set of cytoplasmic and granular interacting proteins (Brinkmann and Zychlinsky, 2012). NETs formed in response to *A. fumigatus* and *C. albicans* infection contain calprotectin, and the presence of this protein in such structures is fundamental for the proper antifungal activity of neutrophils (Urban et al., 2009; McCormick et al., 2010).

The direct effects of zinc deprivation on fungal cells are poorly understood. Assays employing *S. cerevisiae* revealed that, by an unknown mechanism, cells that are exposed to zinc deprivation experience increased levels of ROS (Wu et al., 2007). However, yeast cells employ different strategies to cope with the stress caused by zinc deprivation. As revealed by transcriptomic and functional analyzes in *S. cerevisiae*, low zinc conditions lead to alterations in lipid synthesis, methionine, and sulfate metabolism and to oxidative stress tolerance (Iwanyshyn et al., 2004; Wu et al., 2007, 2009). Furthermore, a genome-wide functional analysis employing a *S. cerevisiae* mutant library that encompasses more than 4500 gene knockout mutants revealed that almost 400 different gene products are necessary for proper growth in zinc-limiting conditions. Among these gene products are those associated with the oxidative stress response, endoplasmic reticulum function, peroxisome biogenesis, histone deacetylation, and zinc uptake (North et al., 2012). Zinc deprivation induced by TPEN also induced accumulation of intracellular ROS in *C. gattii* cells (Schneider et al., 2012). Moreover, proteomic profiling of the dimorphic fungus *Paracoccidioides brasiliensis* exposed to TPEN also revealed an increased expression of proteins involved in stress tolerance, suggesting that zinc deprivation induces stress in these cells (Parente et al., 2013). Thus, it is reasonable to suggest that zinc deprivation hampers fungal development by restricting the activity of zinc-binding proteins and by submitting the fungal cells to different kinds of stress.

## Regulation of zinc homeostasis

The transcriptional responses to zinc deprivation of fungal cells are regulated by the Zap1 transcription factor. The first characterization of the role of Zap1 in regulating zinc homeostasis was performed in *S. cerevisiae* (Zhao and Eide, 1997). This major zinc metabolism regulator contains seven C_2_H_2_ zinc finger domains (ZF). While the domains ZF3–ZF7, located to C-terminal region, are directly associated with the recognition and binding of zinc-responsive elements (ZRE) in the promoters of Zap1-regulated genes, ZF1 and ZF2 lie in the zinc-responsive element present in the activation domain (AD) of this transcription factor. Zinc binding to ZFs in AD2 of Zap1 represses the activity of this transcription factor and therefore inhibits the expression of Zap1-regulated genes (Bird et al., 2003; Herbig et al., 2005). However, zinc can also influence the binding of Zap1 to its ZREs by modulating of the activity of ZF3-ZF7 (Frey et al., 2011). Zap1 activates the expression of more than 60 genes in response to low-zinc environment, including genes that code for zinc transporters (*ZRT1, ZRT2*, ZRT3, *ZRC1*, and *FET4*), proteins related to stress (*CTT1, TSA1*, and *HSP26*), as well as regulating its own expression (Lyons et al., 2000; Wu et al., 2008).

Orthologs of *S. cerevisiae* Zap1 were functionally characterized in three pathogenic fungal species. The *A. fumigatus zafA* expression is induced in zinc-limiting media and repressed by zinc. In addition, null mutants of *zafA* have a reduced ability to grown in low-zinc media as a direct effect of diminished zinc transport. Consequently, such mutants displayed a complete lack of virulence in murine models of aspergillosis. In addition, no conidial germination could be observed in mice infected with mutant cells lacking *zafA* (Moreno et al., 2007).

The *C. albicans* Csr1/Zap1 transcription factor was also shown to influence growth in zinc-deprivation conditions and affect important virulence traits in this pathogenic yeast. While cells lacking the *CSR1/ZAP1* gene displayed reduced filamentation, the same mutants showed increased β-glucan content in their biofilm matrices. Moreover, the biofilms produced by such cells had a predominance cells in the yeast form when compared to wild-type biofilms (Kim et al., 2008; Nobile et al., 2009). The characterization of Csr1/Zap1 targets in biofilm-inducing conditions by chromatin immunoprecipitation analysis showed that this transcription factor recognizes the promoters of approximately 60 genes including the *ZRT1, ZRT2*, and *ZRT3* genes (zinc transporters) and the *CSR1*/*ZAP1* gene itself (Nobile et al., 2009). Further characterization of Csr1/Zap1 revealed that this transcription factor also regulates cell-cell signaling during biofilm development by regulating the expression of Dpp1, a farnesol synthesis protein. Farnesol is an important mediator of biofilm formation as it acts as a quorum-sensing molecule that functions as an inhibitor of the yeast-to-hyphae transition in biofilms (Ganguly et al., 2011).

The Zap1 ortholog from *C. gattii* also regulates the expression of zinc uptake systems. Null mutants of this gene were defective in growth in zinc-deprivation conditions, accumulated intracellular ROS, were hypersensitive to reactive nitrogen species, and displayed attenuated virulence in murine inhalation models of cryptococcosis. Transcriptomic profiling of wild type and *zap1*-null mutants exposed to TPEN revealed that more than 500 genes were differentially expressed between these cell types. The large majority of these differentially expressed gene products were found to be related to adaptive responses to zinc deprivation, but inferred true Zap1 targets were also found including the zinc transporters encoded by *ZIP1* and *ZIP2* (Schneider et al., 2012).

A direct comparison between of *C. albicans, C. gattii*, and *S. cerevisiae* Zap1-dependent transcription profiles revealed a common Zap1 regulon (Table 1). Among positively regulated genes, figure out the genes for the zinc acquisition systems, while a large proportion of negatively regulated genes consist of zinc-binding proteins (Wu et al., 2008; Nobile et al., 2009; Schneider et al., 2012). These proteins include a large family of alcohol dehydrogenases, which are most likely the most abundant zinc-binding proteins in the cell. This trend represents a conserved fungal strategy to shuttle zinc into essential proteins specialized for zinc conservation. This mechanism is characterized by the down-regulation of the expression of abundant zinc storage proteins, such as zinc-dependent alcohol dehydrogenases, in zinc-deprivation conditions. In this way, zinc can easily be mobilized to other zinc-dependent proteins necessary for proper development under these harsh conditions (Eide, 2009).

**Table 1:** **Common set of genes regulated by Zap1 orthologs in yeasts**.

**Functional category**	***S. cerevisiae***		***C. albicans***		***C. gattii***	
	**Gene**	**FC^a^**	**Gene**	**FC^b^**	**Gene**	**FC^c^**
Zinc transport	ZRT1	3.43	ZRT1	4.56	ZIP1 (CNBG_6066)	6.42
	ZRT2	3.43	ZRT2	4.75	ZIP2 (CNAG_2209)	6.96
Zinc conservation	XYL2	−1.32	BZD99	−0.96	CNBG_3878	−2.73
	ADH7	−0.76	AHD6	−0.41	CNBG_2992	−0.81
	ADH1	−1.56	ADH4	−1.19	CNBG_0427	−2.21

a Log_2_ Fold Change values retrieved from microarray expression data of WT and zap1Δ S. cerevisiae cells exposed to low zinc concentrations (61 nM of ZnCl_2_).(Lyons et al., 2000).

b Fold Change values retrieved from microarray expression data of complemented mutant and zap1Δ/ zap1Δ C. albicans biofilms (Nobile et al., 2009).

c Fold Change values retrieved from RNA-Seq expression data of WT and zap1Δ C. gattii cells exposed to a zinc chelator (10 μM of TPEN).(Schneider et al., 2012).

## Conclusions and future studies

Zinc influences diverse mechanisms of fungal pathogenesis by directly associating with virulence determinants (i.e., metalloproteases or Sods) or by regulating the expression of many proteins required for infection. The regulation of zinc acquisition by the Zap1 transcription factors is fundamental for fungal pathogenesis in mammalian hosts. A broader functional characterization of Zip transporters in fungi, including plant and insect fungal pathogens, will elucidate the pivotal role of pathogen zinc-binding proteins during the infectious process. As active zinc deprivation by hosts represents an important antifungal mechanism, development of chelating strategies to control *in vivo* fungal development may be a plausible chemotherapeutic alternative.

### Conflict of interest statement

The authors declare that the research was conducted in the absence of any commercial or financial relationships that could be construed as a potential conflict of interest.
